# AutoPocket2CREST: Automating Binding Pocket Extraction for the CREST Conformer Generation Pipeline

**DOI:** 10.1021/acs.jcim.6c00569

**Published:** 2026-03-23

**Authors:** Christian Fellinger, Marion Sappl, András Szabadi, Benjamin Merget, Klaus-Juergen Schleifer, Thierry Langer

**Affiliations:** † Department of Pharmaceutical Sciences, Faculty of Life Sciences, 27258 University of Vienna, Josef-Holaubek-Platz 2, Vienna 1090, Austria; ‡ Christian Doppler Laboratory for Molecular Informatics in the Biosciences, Department of Pharmaceutical Sciences, University of Vienna, Josef-Holaubek-Platz 2, Vienna 1090, Austria; § Vienna Doctoral School of Pharmaceutical, Nutritional and Sport Sciences (VDS PhaNuSpo), University of Vienna, Josef-Holaubek-Platz 2, Vienna 1090, Austria; ∥ Department of Computational Biological Chemistry, University of Vienna, Währinger Straße 17, Vienna 1090, Austria; ⊥ Vienna Doctoral School in Chemistry (DoSChem), University of Vienna, Währinger Straße 42, Vienna 1090, Austria; # 5184 BASF SE, Carl-Bosch-Strasse 38, Ludwigshafen am Rhein 67056, Germany

## Abstract

AutoPocket2CREST is an automated workflow for preparing protein–ligand binding pockets for CREST conformational sampling. Starting from protein and ligand structures, the method identifies the ligand, constructs a chemically consistent pocket around it, applies optional backbone constraints, and postprocesses CREST conformers to restore structural annotations. AutoPocket2CREST integrates common open-source tools and enables reproducible semiempirical conformational sampling of protein-bound ligands.

## Graphical abstract

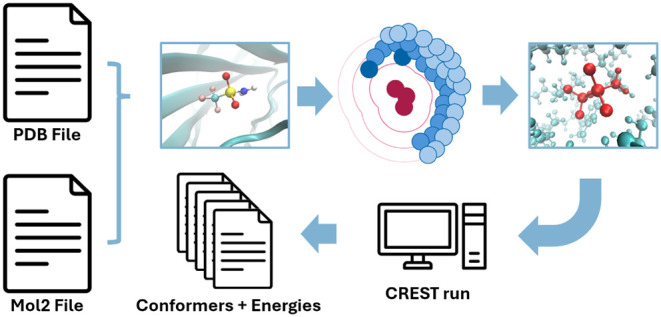

## 1 Introduction

Accurate conformational sampling of protein–ligand systems remains a central challenge in computational chemistry,^1–6^ particularly when quantum-mechanical or semiempirical methods are employed to describe local binding environments.^7^ While molecular dynamics-based approaches are routinely applied to explore protein flexibility, there is growing interest in complementary methods that enable exhaustive conformer generation of ligands and binding-site fragments at reduced computational cost.^8–12^

CREST,^13^ a conformer-rotamer ensemble sampling tool based on the GFN family of semiempirical methods,^14^ has emerged as a powerful approach for exploring molecular conformational space, but also provides the option to use a force-field based approach. Its combination of efficiency, broad chemical coverage, and integration with xTB^15^ makes CREST particularly attractive for applications such as binding-mode refinement, and local investigations of protein–ligand interactions.^16,17^ However, applying CREST directly to protein-bound ligands remains nontrivial. Practical use typically requires manual preparation steps, including ligand identification, binding-site extraction, hydrogenation, charge assignment, constraint definition, and postprocessing of conformer ensembles. These steps are often performed using in-house scripts or interactive tools, limiting reproducibility and hindering wider adoption.

Here, we present AutoPocket2CREST, an automated and reproducible workflow for preparing protein–ligand binding pockets for CREST conformational sampling. It starts from a protein and ligand structure, and constructs a chemically reasonable binding pocket around the ligand, adds missing hydrogen atoms, removes unphysical or disconnected residues, and generates CREST-compatible input files with optional backbone constraints. The workflow further postprocesses CREST conformers to restore residue and atom annotations, enabling integration with established structural biology and cheminformatics tools.

AutoPocket2CREST is implemented as a modular Python package that integrates open-source libraries, including MDAnalysis,^18,19^ RDKit,^20^ Open Babel,^21^ and CREST itself. By automating routine but error-prone preparation steps, the method aims to lower the barrier for applying semiempirical and force-field based conformational sampling to protein–ligand systems and to promote reproducible computational workflows. While AutoPocket2CREST does not replace full protein flexibility treatments, it provides a practical and transparent framework for local binding-site conformational analysis.

## 2 Workflow

Autopocket2CREST can be split into the following seven steps, where the CREST run itself is optional:

1. Setup and Parsing
2. Input Preprocessing
3. Pocket Extraction
4. Hydrogenation
5. Merging and Charge Computation
6. CREST Conformer Search (optional)
7. Cleanup and Reporting

The full pipeline excluding the CREST run itself takes less than 1 s on a workstation with an Intel­(R) Xeon­(R) E-2134 CPU @ 3.50 GHz and a GeForce RTX 2060 for a binding pocket with approximately 200 atoms. The following subsections will go into detail for each step. The Supporting Information contains pseudocode to explain the subsequent sections further.

### 2.1 Setup and Parsing

The first step of the presented tool deals with setup and parsing. Here, a classic argument parser is created to get all information that is required to start the pipeline. The parser expects three mandatory arguments, *protein*_*file*, *ligand*_*file* and *outdir*, where the *ligand*_*file* needs to include the 3D coordinates inside the binding pocket. Both the *protein*_*file* and *ligand*_*file* variables assume that the name or the path to the file is provided. The *outdir* variable is then used to name a subfolder where all the output of this pipeline will be saved.

This parser also provides some optional arguments. The flag −–*no*-*crest* to skip the CREST calculations altogether, and the optional CREST arguments *temp* (default = “310”), *lvl*_*of*_*theory* (default = “gfnff”), and *extra*_*crest*_*arguments* (default = “-squick”) to manipulate the defaults for the CREST conformer search. Afterward, the current directory is requested and saved as a variable as well. All of this is subsequently passed to the *run*_*pipeline*() function to continue with the next steps as described in the enumerated list in the beginning of section 2.

### 2.2 Input Preprocessing

A subfolder *outdir* is created and set as current working directory. First, the function *fix*_*pdb*_*elements* is called with the path to the protein file and “pre_prepared.pdb” as a name for the output. This function guesses the element symbol of each line if it is not already provided in the atom name column. Then the function *filter*_*by*_*altloc*() is called with the previously created file and “prepared.pdb” as output file. This function ensures that only a single position per atom is used in the following steps. This is a necessary step, as alternate states can occur in PDB files. This is done via the inherent structure of a PDB file. It reads every line of a PDB file, checks if the record is an atom line (ATOM or HETATM), keeps the line if its alternate location indicator (altLoc, column 17) is either blank or matches the chosen *keep*_*altloc* (default “A”) and leaves all other lines unchanged.

The next step is to extract the ligand name that will be used as an identifier moving forward. This process has two fallback options. The first step is to extract it directly from the mol2 file from the line immediately following “@<TRIPOS>SUBSTRUCTURE”. If this name is missing or “UNNAMED”, the name that is used in the Mol2 Atoms section is extracted with the same logic as the first check and then used. If for any reason this still does not result in a valid name, a final fallback is executed, where the *ligand*_*name* variable is set to UNNAMED and a corresponding warning for the user is printed.

The prepared PDB file as well as the original mol2 file are then converted to an MDAnalysis Universe, which is used in the next step.

### 2.3 Pocket Extraction

The automated pocket extraction is one of the key parts of the processing pipeline. It aims for a balance between a large enough volume to represent the binding pocket accurately, while also keeping it small enough for maximum efficiency in the following CREST calculation (Figure 1). This logic can be broken down into several steps:

**Figure 1:**
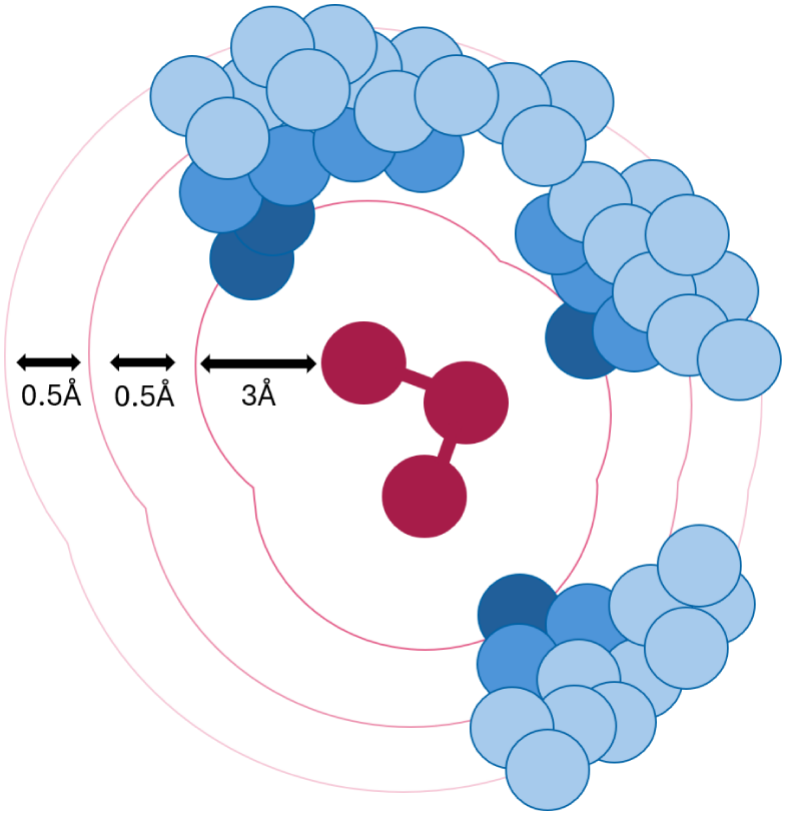
Schematic representation of the iterative nature of the pocket extraction. The first selection only includes all protein atoms that are within 3 Å of the ligand. If the threshold of 70 atoms is not reached, this range is increased by 0.5 Å or until 50 iterations are reached.

Step 1 checks if the ligand is present and if it has a reasonable size. 120 atoms was chosen as a threshold, since a bigger ligand molecule will often lead to binding pockets that include more atoms than CREST can support, due to inherent size limitations of approximately 500 atoms. Step 2 selects all protein atoms that are within 3 Å of the ligand. If this leads to less than 70 atoms, this radius is increased iteratively by 0.5 Å steps until enough atoms are found, or 50 attempts are made. These atoms are then extended to select their full residues. This leads to a preliminary pocket, which is then extended by 2.6 Å while excluding ligand atoms and any remaining water to include the *N*-methyl and acetyl groups at the ends of the residues to make it chemically more similar to a full protein pocket. Since this step can lead to some isolated atoms around the actual pocket, an additional cleaning step removes isolated atoms that are not connected to any others within 1.9 Å. The fully prepared pocket is then saved as “test_pocket_extended.pdb”.

### 2.4 Hydrogenation

The cleaning step in the previous section can still lead to edge cases where more than a single atom is being kept and considered undesirable. To prevent this and to make sure that the protonation of the pocket is valid, Open Babel is used as a first step to remove all hydrogens and generate connectivity information. The dehydrogenation is necesarry to ensure correct hydrogen placement after the removal of unconnected segments. A second cleanup function parses a PDB file to collect atom identifiers (ATOM, HETATM) and connectivity information (CONECT statements), notes which residues are bonded to others, keeps only residues that have at least one covalent link to another residue, and writes a clean PDB containing only connected residues and valid CONECT statements. The preceding dehydrogenation is necessary to ensure correct hydrogen placement after the removal of unconnected segments. The cleaned PDB file is then processed again by Open Babel to protonate the pocket at a pH of 7.4, which leads to a fully cleaned and reasonable pocket structure.

### 2.5 Merging and Charge Computation

This final pocket is once again translated to an MDAnalysis universe object and then merged with the ligand universe, which is then saved as a final PDB file to be used in the remaining steps of the workflow. It is also used to calculate the formal charge of the full system by the RDKit Chem module.

### 2.6 CREST Conformer Search

This part of the pipeline can be turned off with the “–no-crest” flag. If enabled (default) this step starts by reading in the final PDB file and generating a list of all indices of all atoms that are not part of the ligand. This list and the path to the PDB file is then used to generate the necessary constraints. It starts by compressing the list of atoms that are supposed to be constrained. A function detects continuous integer sequences and converts it to a compact range list to ensure compatibility with CREST. e.g., it takes the list “1,2,3,4,5,6,8,9,10” and converts it to “1–6,8–10”. Afterward, it calls CREST with “–constrain” to generate the constraint file, and then renames the default output file “.xcontrol.sample” to “constraints.inp”.

Having all of this required information at hand, the actual CREST run can be started. The CREST execution command line is built-up and executed. The selected default values are the same as in the parsing step and will be overwritten if the user chooses to use different values.

### 2.7 Cleanup and Reporting

The first step is to convert the resulting CREST conformers from an xyz to a PDB file using Open Babel. To mitigate information loss, the following functions are used to transfer ligand residue information.

These functions extract the atom data of the template PDB, read all conformers from the output of CREST, update each conformer with the metadata of the template and finally write the updated conformers to the output file. The first function call extracts the atomic information from a reference PDB template file as shown in the Supporting Information.

The second function call reads in the multi-PDB that Open Babel generated from the CREST xyz file and splits it up into individual conformers by the “MODEL”/“ENDMODEL” statements and returns a list as shown below.

The final function call replaces the atom name, residue name and residue number in a conformer with the information previously extracted from the reference PDB.

Combining all of these procedures results in a final multi-PDB file called “crest_conformers_updated.pdb” with the residue information on the reference PDB file.

Finally, a simple helper function is used to delete temporary files.

## 3 Usage

AutoPocket2CREST is designed as a command line tool for Linux. The full code can be found on GitHub (https://github.com/molinfo-vienna/autopocket2crest) and cloned on the local machine. To install, it is recommended to use either Conda or Mamba and create an environment with the following commands:
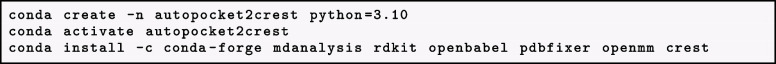


Alternatively, the provided environment.yml file can be used:
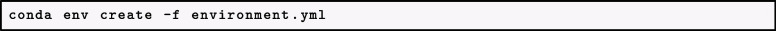


Install autopocket2crest from within the cloned repository:
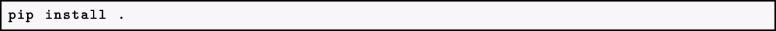


To use AutoPocket2CREST, use the following command
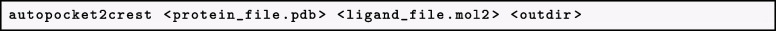


where <*protein*_*file*.*pdb*> is the path to the protein file, <*ligand*_*file*.*mol*2> is the path to the ligand file, and <*outdir*> is a name for a working directory that will be created in the current working directory to save all results. Optional keywords and functionalities are available in the AutoPocket2Crest help (-h) or in the Supporting Information.

See Table 1 for the most important output of a successful run.

**Table 1:** Output Files of AutoPocket2CREST

crest_conformers_updated.pdb	→ contains the conformers with transcribed ligand residue information in PDB format
crest_conformers.pdb	→ contains the conformers without transcribed ligand residue information in PDB format
crest_conformers.xyz	→ contains the conformers in xyz format
crest.out	→ contains the output of the CREST run

## 4 Conclusion

Preparing chemically consistent protein–ligand pockets for conformational sampling remains a time-consuming and error-prone task, often requiring extensive manual intervention and expert knowledge. In this work, we presented AutoPocket2CREST, an automated and modular workflow that extracts ligand-centered binding pockets, prepares defined structures, and interfaces seamlessly with CREST for conformational exploration.

By combining automatic pocket construction, hydrogenation, charge determination, and constraint generation into a single reproducible pipeline, AutoPocket2CREST significantly lowers the technical barrier to applying a wide range of CREST levels of theory to protein–ligand systems. The workflow is designed to require minimal user input while remaining transparent and customizable, enabling straightforward integration into existing computational chemistry pipelines.

We hope that AutoPocket2CREST will help to streamline conformational sampling in structure-based drug design and related applications. Owing to its modular design, the workflow can be readily extended and improved upon, providing a flexible foundation for future methodological developments.

## 5 Data and Software Availability

The tool can be downloaded via its GitHub repository (https://github.com/molinfo-vienna/autopocket2crest) free of charge.

## Supplementary Material



## Notes

SI file contains a more in-depth explanation of the logic of this tool by supplying pseudocode for each used function (PDF)
