# Supplementary material for: AutoPocket2CREST: Automating Binding Pocket Extraction for the CREST Conformer Generation Pipeline
Source: J Chem Inf Model. 2026 Mar 23;66(7):3432–6. doi: 10.1021/acs.jcim.6c00569 (PMC13080960; doi:10.1021/acs.jcim.6c00569)
Supplement: Supplementary file 1 [file ci6c00569_si_001.pdf]

Supporting Information

AutoPocket2CREST: Automating binding Pocket Extraction for the CREST Conformer Generation Pipeline

Christian Fellinger[1,2,3*], Marion Sappl[4,5], András Szabadi[4], Benjamin Merget[6], Klaus-Juergen Schleifer[6], Thierry Langer[1,2]

[1]Department of Pharmaceutical Sciences, Faculty of Life Sciences, University of Vienna, Josef-Holaubek-Platz 2, 1090 Vienna, Austria.
[2]Christian Doppler Laboratory for Molecular Informatics in the Biosciences, Department of Pharmaceutical Sciences, University of Vienna, Josef-Holaubek-Platz 2, 1090 Vienna, Austria.
[3]Vienna Doctoral School of Pharmaceutical, Nutritional and Sport Sciences (VDS PhaNuSpo), University of Vienna, Josef-Holaubek-Platz 2, 1090 Vienna, Austria.
[4]Department of Computational Biological Chemistry, University of Vienna, Währinger Straße 17, 1090 Vienna, Austria.
[5]Vienna Doctoral School in Chemistry (DoSChem), University of Vienna, Währinger Straße 42, 1090 Vienna, Austria.
[6]BASF SE, Carl-Bosch-Strasse 38, 67056 Ludwigshafen am Rhein, Germany.

*Corresponding author(s). E-mail(s): christian.fellinger@univie.ac.at,christian@fellingers.net;
Contributing authors: marion.sappl@univie.ac.at; andras.szabadi@univie.ac.at; benjamin.merget@basf.com; klaus-juergen.schleifer@basf.com; thierry.langer@univie.ac.at;

1 AutoPocket2CREST Pseudocode

This Supplementary information aims to explain the functions mentioned in the Application Note in more detail using pseudocode. For this purpose, the same structure as in the main text was chosen as seen below.

1. Setup and Parsing
2. Input Preprocessing
3. Pocket Extraction
4. Hydrogenation
5. Merging and Charge Computation
6. CREST Conformer Search (optional)
7. Cleanup and Reporting

These steps are executed by calling the function *run_pipeline*() which combines different helper functions for generating the desired files. The following subsections will provide further detail about each of these steps and list the necessities of each. The full code can be found on GitHub (https://github.com/molinfo-vienna/autopocket2crest).

1.1 Setup and Parsing

The first step of the presented tool deals with setup and parsing. Here, a classic argument parser is created to get all information that is required to start the pipeline. The parser expects three mandatory arguments:

```
ADD required argument: "protein_file"  -> Protein PDB input file
ADD required argument: "ligand_file"   -> Ligand MOL2 input file
ADD required argument: "outdir"         -> ID for naming the output folder
```

Both the *protein_file* and *ligand_file* variables assume that the name or the path to the file is provided. The *outdir* variable is then used to name a subfolder where all the output of this pipeline will be saved.

This parser also provides some optional arguments:

```
ADD optional flag: "--no-crest"        -> If present, skip CREST execution

ADD optional argument: "temp"           (default = "310")
    -> Temperature setting for CREST runs (in Kelvin)
ADD optional argument: "lvl_of_theory"  (default = "gfnff")
    -> Level of theory used by CREST
ADD optional argument: "extra_crest_args" (default = "-squick")
    -> Additional user-specified arguments for CREST execution
```

These are used to manipulate the defaults for the CREST conformer search, or to skip it altogether. Afterwards the current directory is requested and saved as a variable as well. All of this is subsequently passed to the *run_pipeline*() function to continue with the next steps as described in the enumerated list in section ??.

1.2 Input Preprocessing

The parsed *outdir* is used to generate a subfolder with this name, and the current working directory is changed to this as well. First, the function *fix_pdb_elements* is called with the path to the protein file and "pre_prepared.pdb" as a name for the output. This function guesses the element symbol of each line if it is not already provided by inferring them from the atom name column as shown below:

```
FUNCTION fix_pdb_elements(input_pdb, output_pdb):
    PURPOSE:
        Ensure that every ATOM/HETATM record in a PDB file
        has a valid element identifier (columns 77-78).
        Missing elements are inferred from the atom name.

    # --- Helper function to infer element from atom name ---
    FUNCTION infer_element(atom_name):
        REMOVE digits and spaces from atom_name -> name

        IF name is empty:
            RETURN "  "   # blank element

        IF name has at least two letters AND second letter is lowercase:
            RETURN capitalized first two letters    # e.g. "Cl", "Fe"
        ELSE:
            RETURN capitalized first letter         # e.g. "C", "N", "O"
    END FUNCTION

    OPEN input_pdb for reading AS fin
    OPEN output_pdb for writing AS fout

    FOR each line IN fin:
        IF line starts with "ATOM" OR "HETATM":

            # Ensure line is long enough to contain element field
            padded_line <- line without newline, padded to at least 78 characters

            # Extract existing element field (columns 77-78)
            existing_element <- padded_line[76-78].strip()

            IF existing_element is not empty:
                WRITE original line to fout
            ELSE:
                atom_name <- padded_line[12-16]
                element <- infer_element(atom_name)

                fixed_line <-
                    padded_line[1-76] +
                    right-aligned element in columns 77-78 +
                    padded_line[79-end]

                WRITE fixed_line + newline to fout
        ELSE:
            WRITE line unchanged to fout
    END FOR

    CLOSE fin
    CLOSE fout
END FUNCTION
```

Then the function *filter_by_altloc*() is called with the path to the protein file and "prepared.pdb" as a name for the file that it creates:

```
FUNCTION filter_pdb_by_altloc(input_file, output_file, keep_altloc = 'A'):
    PURPOSE:
        Keep only atoms with the desired alternate location (altLoc)
```

```
        identifier from a PDB file, and write the filtered structure
        to a new file.

    OPEN input_file for reading AS fin
    OPEN output_file for writing AS fout

    FOR each line in fin:
        IF line starts with "ATOM" OR "HETATM":
            altloc <- character at position 17 of the line (index 16)

            IF altloc is a space OR altloc equals keep_altloc:
                WRITE line to fout
            END IF
        ELSE:
            # Copy all non-atom lines (e.g., headers, TER, END)
            WRITE line to fout
        END IF
    END FOR

    CLOSE both files
END FUNCTION
```

This function ensures that only a single position per atom is used in the following steps. This is a necessary step, since often the experimental PDB files provide two different interpretations of parts of the protein structure. This is done via the inherent structure of a PDB file. It reads every line of a PDB file, checks if the record is an atom line (ATOM or HETATM), keeps the line if its alternate location indicator (altLoc, column 17) is either blank or matches the chosen *keep_altloc* (default 'A') and leaves all other lines unchanged.

The next step is to extract the ligand name that will be used as an identifier moving forward. This process has two fallback options as shown in this pseudo code:

```
FUNCTION get_ligand_name(mol2_file, pdb_file):
    PURPOSE:
        Identify the ligand residue name using a MOL2 file,
        with fallback strategies using the PDB and MOL2 atom records.

    DEFINE typical_cofactor_names AS set of:
        - common cofactors
        - solvents
        - ions
        - metals
        - elements

    ligand_name <- NULL

    # --- Step 1: Try MOL2 SUBSTRUCTURE section ---
    OPEN mol2_file FOR reading
    next_line <- FALSE
    FOR each line IN mol2_file:
        IF next_line is TRUE:
            ligand_name <- second column of line
            BREAK
        ELSE IF line starts with "@<TRIPOS>SUBSTRUCTURE":
            next_line <- TRUE
    CLOSE mol2_file

    # --- Step 2: Fallback - MOL2 ATOM section ---
    IF ligand_name is NULL OR ligand_name == "UNNAMED":
        OPEN mol2_file FOR reading
        next_line <- FALSE
        FOR each line IN mol2_file:
            IF next_line is TRUE:
                parts <- split line into fields
```

```
                IF number of fields > 7:
                    ligand_name <- field 8
                    BREAK
            ELSE IF line starts with "@<TRIPOS>ATOM":
                next_line <- TRUE
        CLOSE mol2_file

    # --- Step 3: Ultimate fallback ---
    IF ligand_name is still NULL:
        PRINT warning message
        ligand_name <- "UNNAMED"

    RETURN ligand_name
END FUNCTION
```

The first step is to extract it directly from the mol2 file from the line immediately following ”@<TRIPOS>SUBSTRUCTURE”, if this name is missing or ”UNNAMED”, the name that is used in the Mol2 Atoms section is extracted with the same logic as the first check and then used. If for any reason this still does not result in a valid name, a final fallback is executed, where the *ligand_name* variable is just set to UNNAMED and a corresponding warning for the user is printed.

Then the prepared PDB file as well as the original mol2 file is converted into an MDAnalysis Universe, which is used in the next step.

1.3 Pocket Extraction

The automated pocket extraction is one of the key parts of the processing pipeline. It needs to find a balance between a pocket that is large enough to represent the binding pocket accurately, while also keeping it small enough for the following CREST calculation. The logic can be seen below:

```
FUNCTION cut_pocket(ligand, ligand_resname, universe):

    # --- Step 1: Validate ligand selection ---
    IF number of atoms in ligand == 0 THEN
        PRINT "Warning: No ligand found with residue name {ligand_resname}. Exiting
    ."
        EXIT program
    ELSE IF number of atoms in ligand > 120 THEN
        PRINT "Warning: Ligand has more than 120 atoms. This might be an error.
    Exiting."
        EXIT program
    ELSE
        PRINT "Ligand found with {number of ligand atoms} atoms."
    END IF


    # --- Step 2: Initial pocket selection ---
    PRINT "Selecting pocket atoms..."
    pocket <- select atoms within 3 angstrom of ligand (first shell)
    PRINT "Initial pocket atoms:", number of atoms in pocket


    # --- Step 3: Adjust pocket radius if too few atoms found ---
    i <- 0
    WHILE number of atoms in pocket < 70 DO
        PRINT "No atoms found in pocket. Increasing radius..."
        i <- i + 0.5
        pocket <- select atoms within (3 + i) angstrom of ligand
        IF i > 50 THEN
            PRINT "No atoms found after 50 attempts. Exiting."
```

```
            EXIT program
        ELSE
            PRINT "Found pocket atoms after", i * 2, "iterations with radius", 3 + i, "angstrom"
            PRINT "New pocket atom count:", number of atoms in pocket
        END IF
    END WHILE


    # --- Step 4: Include full residues for pocket atoms ---
    pocket <- all atoms belonging to residues that contain pocket atoms


    # --- Step 5: Expand pocket and exclude ligand ---
    pocket_extended <- select atoms that are:
        (in pocket_group) OR (within 2.6 angstrom of pocket_group AND not in ligand_group)

    PRINT "Extended pocket atoms:", number of atoms in pocket_extended


    # --- Step 6: Exclude ligand and water molecules ---
    pocket_extended <- select atoms from pocket_extended
                        where residue name =/= ligand_resname AND =/= HOH


    # --- Step 7: Remove isolated atoms (no neighbors within 1.9 angstrom) ---
    isolated_atoms <- empty list
    FOR each atom_i in pocket_extended:
        atom_group <- that single atom
        test_group <- select atoms within 1.9 angstrom of atom_group
        IF test_group is empty THEN
            PRINT "Warning: Atom", atom_i, "is isolated. Removing it."
            ADD atom_i to isolated_atoms
        END IF
    END FOR

    FOR each atom_j in isolated_atoms:
        pocket_extended <- pocket_extended excluding atom_j
    END FOR


    # --- Step 8: Write final pocket structure to file ---
    WRITE pocket_extended to "test_pocket_extended.pdb"
    PRINT "Pocket size:", number of atoms in pocket_extended

END FUNCTION
```

Step 1 checks if the ligand is present and if it has a reasonable size. 120 Atoms was chosen as a threshold, since a bigger ligand molecule will often lead to binding pockets that include more atoms than CREST can support. Step 2 selects all protein atoms that are within 3 Å of the ligand. If this leads to less than 70 Atoms, this radius is increased iteratively by 0.5 Å steps until enough atoms are found, or 50 attempts are made. These atoms are then extended to select their full residues. This leads to a preliminary pocket, which is then extended by 2.6 Å while excluding ligand atoms and any remaining water to include the N-Methyl and Acetyl groups at the ends of the residues to make it chemically more similar to a full protein pocket. Since this step can lead to some isolated atoms around the actual pocket, an additional cleaning step removes isolated atoms that are not connected to any others within 1.9 Å. The fully prepared pocket is then saved as ”test_pocket_extended.pdb”.

1.4 Hydrogenation

The cleaning step in the previous section can still lead to edge cases where more than a single atom is being kept and considered undesirable. To prevent this and to make sure that the protonation of the pocket is valid, obabel is used as a first step to remove all hydrogens and generate connectivity information. The following second cleanup step is applied on top:

```
FUNCTION remove_unbonded(pdb_file, output_file):
    PURPOSE:
        Remove all residues in a PDB structure that are not covalently bonded
        to any other residue, based on CONECT records.

    # --- Step 1: Read PDB file ---
    READ all lines from pdb_file INTO list lines

    INITIALIZE:
        atom_info      <- empty dictionary   # maps atom_serial -> (residue_id, line)
        residue_atoms  <- empty dictionary   # maps residue_id -> set of atom_serials
        conect_map     <- empty dictionary   # maps atom_serial -> set of bonded atom_serials


    # --- Step 2: Parse atom and CONECT information ---
    FOR each line IN lines:
        IF line starts with "ATOM" OR "HETATM":
            res_name <- characters 18-20, trimmed
            chain_id <- character 22
            res_seq  <- integer from characters 23-26
            res_id   <- tuple (chain_id, res_name, res_seq)

            atom_serial <- integer from characters 7-11

            STORE atom_info[atom_serial] = (res_id, line)
            ADD atom_serial to residue_atoms[res_id] (create set if missing)

        ELSE IF line starts with "CONECT":
            a1 <- integer from characters 7-11
            bonded_list <- atoms listed in positions 12-31 (each 5 chars)
            FOR each a2_str IN bonded_list:
                IF a2_str is not empty:
                    a2 <- integer(a2_str)
                    ADD a2 to conect_map[a1]
                    ADD a1 to conect_map[a2]    # ensure bidirectional connectivity
        END IF
    END FOR


    # --- Step 3: Identify residues connected to other residues ---
    residues_to_keep <- empty set

    FOR each (atom_serial, (res_id, _)) in atom_info:
        FOR each bonded_atom IN conect_map[atom_serial]:
            bonded_res <- atom_info[bonded_atom].residue_id
            IF bonded_res exists AND bonded_res =/= res_id:
                ADD res_id to residues_to_keep
                BREAK inner loop
        END FOR
    END FOR


    # --- Step 4: Write filtered output PDB ---
    OPEN output_file for writing AS fout

```

```
    FOR each line IN lines:
        IF line starts with "ATOM" OR "HETATM":
            atom_serial <- integer from characters 7-11
            res_id <- atom_info[atom_serial].residue_id
            IF res_id is an element of residues_to_keep:
                WRITE line to fout

        ELSE IF line starts with "CONECT":
            TRY:
                a1 <- integer from characters 7-11
                bonded <- list of integers found in columns (12-31)
                all_atoms <- [a1] + bonded
                IF all atoms in all_atoms belong to residues_to_keep:
                    WRITE line to fout
            EXCEPT ValueError:
                CONTINUE
        ELSE:
            # Copy non-atom, non-conect lines as-is (e.g., HEADER, END)
            WRITE line to fout
        END IF
    END FOR
    CLOSE fout


    PRINT "Removed unbonded residues. Output:", output_file
    RETURN output_file
END FUNCTION
```

This function parses a PDB file to collect atom identifiers (ATOM, HETATM) and connectivity information (CONECT statements), notes which residues are bonded to others, keeps only residues that have at least one covalent link to another residue, and writes a clean PDB containing only connected residues and valid CONECT statements. This only works because of the previous patching step. The cleaned PDB file is then processed again by obabel to protonate the pocket at a pH of 7.4, which leads to a fully cleaned and reasonable pocket structure.

1.5 Merging and Charge Computation

This final pocket is once again translated to an MDAnalysis universe object and then merged with the ligand universe, which is then saved as a final PDB file to be used in the remaining steps of the workflow. It is also used to calculate the formal charge of the full system by the RDKit Chem module.

1.6 CREST Conformer Search

This part of the pipeline can be turned off with the ”–no-crest” flag. If enabled (default) this step starts by reading in the final PDB file and generating a list of all indices of all atoms that are not part of the ligand. This list and the path to the PDB file is then used by the following function to generate the necessary constraints.

```
FUNCTION generate_constraints(pdb_file, backbone_indices):
    PURPOSE:
        Create a constraint file ("constraints.inp") for CREST
        based on the atom indices of the protein backbone.

    # --- Step 1: Compress backbone indices into range strings ---
    ranges <- CALL compress_ranges(backbone_indices)
    constrain_str <- join all elements of ranges using commas
                     (e.g., "1-5,8-10,12")
```

```
    # --- Step 2: Run CREST to generate constraint template ---
    EXECUTE shell command:
        "crest [pdb_file] --constrain [constrain_str]"

    # --- Step 3: Rename output file if CREST produced one ---
    IF file ".xcontrol.sample" exists:
        RENAME ".xcontrol.sample" -> "constraints.inp"

    PRINT "Constraint file created."

    RETURN "constraints.inp"
END FUNCTION
```

It starts by compressing the list of atoms that are supposed to be constrained by calling the function below. Afterward, it calls CREST with ”–constrain” to generate the constraint file, and then renames the default output file ”.xcontrol.sample” to ”constraints.inp”.

```
FUNCTION compress_ranges(indices):
    PURPOSE:
        Convert a list of integer indices into a compact list of
        continuous ranges for easier reading and use in constraint files.

    IF indices list is empty:
        RETURN empty list

    # --- Step 1: Sort and remove duplicates ---
    indices <- sorted unique values of indices

    # --- Step 2: Initialize range tracking variables ---
    start <- indices[0]
    prev  <- indices[0]
    ranges <- empty list

    # --- Step 3: Iterate through remaining indices ---
    FOR each n IN indices[1:]:
        IF n == prev + 1:
            # Still inside a continuous range
            prev <- n
        ELSE:
            # End of current range; append formatted range string
            IF start =/= prev:
                ADD string "{start}-{prev}" to ranges
            ELSE:
                ADD string "{start}" to ranges
            END IF
            # Start new range
            start <- n
            prev <- n
        END IF
    END FOR

    # --- Step 4: Append the final range ---
    IF start =/= prev:
        ADD string "{start}-{prev}" to ranges
    ELSE:
        ADD string "{start}" to ranges
    END IF

    RETURN ranges
END FUNCTION
```

The function detects continuous integer sequences and converts it to a compact range list for further use. E.g. it takes the list ”1,2,3,4,5,6,8,9,10” and converts it to

”1-6,8-10”. This is important because CREST is not able to parse a list that is too long.

Having all of this required information at hand the actual CREST run can be started. The following pseudo code shows how the CREST execution command line is built-up and is then executed. The selected default values are the same as in the parsing step and will be overwritten if the user chooses to use different values.

```
FUNCTION run_crest(xyz_file, constraint_file = None,
                   charge = 0, temp = "310",
                   lvl_of_theory = "gfnff",
                   extra_crest_args = "-squick"):

    PURPOSE:
        Run a CREST conformer search using specified parameters.
        Optionally include a constraint file if available.

    # --- Step 1: Build base CREST command ---
    cmd <- "crest " + xyz_file
    cmd <- cmd + " -" + lvl_of_theory                # e.g., "-gfnff"
    cmd <- cmd + " -chrg " + charge                  # molecular charge
    cmd <- cmd + " -gbsa h2o "                       # implicit solvent model
    cmd <- cmd + extra_crest_args                    # additional CREST flags
    cmd <- cmd + " --temp " + temp                   # temperature in Kelvin
    cmd <- cmd + " --legacy"                         # legacy mode for
     compatibility

    # --- Step 2: Add constraint file if provided ---
    IF constraint_file exists:
        cmd <- cmd + " -cinp " + constraint_file

    # --- Step 3: Redirect output to file ---
    cmd <- cmd + " > crest.out"

    # --- Step 4: Execute the CREST command ---
    PRINT "Executing:", cmd
    EXECUTE shell command(cmd)

    # --- Step 5: Return the expected output filename ---
    RETURN "crest.out"
END FUNCTION
```

After finishing the CREST conformer calculation, some cleanup and reporting is in order.

1.7 Cleanup and Reporting

The first step is to convert the resulting CREST conformers from a xyz to an PDB file using openbabel. Of course, this leads to some information loss. To mitigate that, the following functions are used to at least transfer the ligand residue information.

```
FUNCTION transfer_pdb_info(template_path, conformers_path, output_path):
    PURPOSE:
        Synchronize conformer PDB atom and residue information
        with a reference template structure.

    # --- Step 1: Extract template atom data ---
    template_data <- CALL get_template_fields(template_path)

    # --- Step 2: Read all conformers from CREST output ---
    conformers <- CALL parse_conformers(conformers_path)

    # --- Step 3: Update each model with template metadata ---
```

```
    updated <- empty list
    FOR each model IN conformers:
        new_model <- CALL update_model(model, template_data)
        EXTEND updated WITH new_model
    END FOR

    # --- Step 4: Write all updated models to output file ---
    OPEN output_path for writing AS f
    WRITE all lines from updated INTO f
    CLOSE f

    PRINT "Updated conformers written to", output_path

    RETURN output_path
END FUNCTION
```

This function extracts the atom data of the template PDB, reads all conformers from the output of CREST, updates each conformer with the metadata of the template and finally writes the updated conformers to the output file. The first function call extracts the atomic information from a reference PDB template file as shown below:

```
FUNCTION get_template_fields(template_file):
    PURPOSE:
        Extract atomic field information (atom name, residue name, residue number)
        from a reference PDB template file.

    data <- empty list

    OPEN template_file for reading AS f
    FOR each line in f:
        IF line starts with "ATOM" OR "HETATM":
            atom_name <- characters 13-16
            res_name  <- characters 18-20
            res_num   <- characters 23-26
            APPEND (atom_name, res_name, res_num) TO data
        END IF
    END FOR
    CLOSE f

    RETURN data
END FUNCTION
```

The second function call reads in the multi-PDB that openbabel generated from the CREST xyz file and splits them up into individual conformers by the ”MODEL”/”ENDMODEL” statements and returns a list of them as shown below.

```
FUNCTION parse_conformers(conformers_file):
    PURPOSE:
        Read a multi-model PDB file and separate it into individual conformers.
        If no "MODEL"/"ENDMDL" blocks are found, treat entire file as one conformer
    .

    conformers <- empty list
    current <- empty list
    inside <- FALSE

    OPEN conformers_file for reading AS f
    FOR each line in f:
        IF line starts with "MODEL":
            current <- [line]
            inside <- TRUE

        ELSE IF line starts with "ENDMDL":
            APPEND line to current
            APPEND current to conformers
```

```
            inside <- FALSE

        ELSE IF inside is TRUE:
            APPEND line to current
        END IF
    END FOR
    CLOSE f

    # --- Fallback: handle files without explicit MODEL blocks ---
    IF conformers is empty:
        PRINT "Warning: No models found, treating file as single conformer."

        conf <- ["MODEL        1\n"]
        OPEN conformers_file for reading AS f
        FOR each line in f:
            IF line starts with "ATOM" OR "HETATM":
                APPEND line to conf
        END FOR
        CLOSE f
        APPEND "ENDMDL\n" to conf
        APPEND conf to conformers

    RETURN conformers
END FUNCTION
```

The final function call replaces the atom name, residue name and residue number in a conformer with the information previously extracted from the reference PDB.

```
FUNCTION update_model(model_lines, template_data):
    PURPOSE:
        Replace atom name, residue name, and residue number in a conformer
        with those from a reference template PDB.

    out <- empty list

    FOR i, line IN model_lines with index i:
        IF line starts with "ATOM" OR "HETATM" AND i < length(template_data):
            (atom_name, res_name, res_num) <- template_data[i]

            new_line <-
                line[1-12] + atom_name +
                line[17] + res_name +
                line[21-22] + res_num +
                line[27-end]

            APPEND new_line to out
        ELSE:
            APPEND line to out
        END IF
    END FOR

    RETURN out
END FUNCTION
```

Combining all of these procedures results in a final multi-PDB file called ”crest_conformers_updated.pdb” with the residue information of the reference PDB file.

Finally, a simple helper function is used to delete some unnecessary files that were created during the processing steps:

```
FUNCTION cleanup_temp_files(file_list):
    PURPOSE:
        Delete intermediate or temporary files created during the
        AutoPocket2CREST pipeline to keep the workspace clean.

```

```
    FOR each file_path IN file_list:
        IF file_path exists on disk:
            TRY:
                DELETE file_path
                PRINT "Removed: [file_path]"
            EXCEPT any error AS e:
                PRINT "Could not remove [file_path]: [error message]"
        END IF
    END FOR
END FUNCTION
```
